# Validation of a Liquid–Liquid Extraction Method to Study the Temporal Production of D-Series Resolvins by Head Kidney Cells from Atlantic Salmon (*Salmon salar*) Exposed to Docosahexaenoic Acid

**DOI:** 10.3390/molecules28124728

**Published:** 2023-06-13

**Authors:** Pedro Araujo, Sarah Iqbal, Aleksander Arnø, Marit Espe, Elisabeth Holen

**Affiliations:** 1 Institute of Marine Research (HI), P.O. Box 1870 Nordnes, N-5817 Bergen, Norway; sarah.iqbal@hi.no (S.I.); aleksander.arno@student.uib.no (A.A.); marit.espe@hi.no (M.E.); elisabeth.holen@hi.no (E.H.); 2 Department of Chemistry, University of Bergen, Allégaten 41, N-5007 Bergen, Norway; 3 Department of Biological Sciences, University of Bergen, Thormøhlens Gate 53A, N-5006 Bergen, Norway

**Keywords:** resolvins, liquid chromatography quadrupole mass spectrometry, Atlantic salmon, head kidney cells, liquid–liquid extraction

## Abstract

A simple and rapid method for the extraction of D-series resolvins (RvD1, RvD2, RvD3, RvD4, RvD5) released into Leibovitz’s L-15 complete medium by head kidney cells from Atlantic salmon and the further determination of liquid chromatography triple quadrupole mass spectrometry is proposed. A three-level factorial design was proposed to select the optimal concentrations of internal standards that were used in the evaluation of the performance parameters, such as linear range (0.1–50 ng mL^−1^), limits of detection and quantification (0.05 and 0.1 ng mL^−1^, respectively), and recovery values ranging from 96.9 to 99.8%. The optimized method was used to determine the stimulated production of resolvins by head kidney cells exposed to docosahexaenoic acid, and the results indicated that it is possible that the production was controlled by circadian responses.

## 1. Introduction

Resolvins from the D-series are bioactive oxygenated metabolites of docosahexaenoic acid (22:6n-3; DHA) that were discovered in mice exudate cells treated with aspirin and DHA and were termed resolvins due to their role in dampening and promoting the resolution of inflammation processes [1]. Resolvins from the D-series (RvD) are biosynthesized by the action of 15-LOX on DHA to produce 17S-hydroperoxydocosahexaenoic acid that is converted to various types of resolvin D (RvD): RvD_1,_ RvD_2,_ RvD_3_, RvD_4_, RvD_5_ and RvD_6_ by the action of 5-LOX [1,2]. RvD_1_ has therapeutic effects such as slowing the progression of osteoarthritis in joints, preventing neuronal dysfunction in Parkinson’s disease, and increasing efferocytosis in the elderly [3]. RvD_2_ promotes subcellular localized healing, and regenerative and protective effects in burn wounds, such as keratinocyte restoration, muscle regeneration, tissue necrosis restriction, tumor growth inhibition and clearing cellular debris in mice [3,4]. RvD_3_ has powerful anti-inflammatory effects on leukocytes, decreases the levels of pro-inflammatory cytokines (MCP-1, IL-6, and keratinocyte chemoattractant protein), and eicosanoids (LTB_4_, PGD_2_ and TxB_2_), inhibits neutrophil transmigration and enhances macrophage absorption of microbial particles [5]. RvD_4_ protects organs in cases of ischemia kidney injury [3,6]. RvD_5_ improves phagocytosis by increasing neutrophil and macrophage movement, modulates TNF-α and NF-κB in blood, synovial fluid and exudates discharged after a hemorrhage, and, in the late stages of coagulation, prolongs the agglutination process and reduces the likelihood of bleeding [7]. RvD_6_ increases nerve regeneration and stimulates hepatocyte growth factor genes specifically as upstream regulators and a gene network involved in axon growth and the suppression of neuropathic pain, indicating a novel function of this lipid mediator to maintain cornea integrity and homeostasis after injury [2,8].

In general, resolvins produced from DHA have demonstrated promising therapeutic advantages in terms of cell damage reduction, oxidative stress inhibition, and tumor growth suppression [9]. In mammals, there is growing evidence that resolvins may assist in the resolution of acute inflammation and potently suppress inflammatory and neuropathic pain. Although it is unknown whether this is the case in fish, a study conducted by Ruyter and colleagues showed that greater dietary DHA levels lead to higher concentrations of resolvins in plasma, which may have health benefits in fish [10]. Neuronal deficiencies and developmental issues in larvae have been reported in fish fed with a diet scarce in DHA [11], hence the possible link between resolvins in the neuronal function of fish. 

Different approaches have been reported for the determination of resolvins in different kinds of samples. Enzyme immunoassay (EIA) kits have been used for determining the production of RvD_1_ in human and fish plasma [10,12] with high sensitivity. However, it is also well known that EIA is prone to cross-reactivity, which in turn causes an overestimation of the levels of specific resolvins. In addition, EIA are limited to just one type of resolvin per commercial kit (e.g., either RvD_1_ or RvD_2_, which makes the technique remarkably expensive when different resolvins are considered. 

High performance liquid chromatography with UV diode array detection (HPLC-DAD) and gas chromatography coupled to mass spectrometry (GC-MS) have been used as alternative techniques to validate the results of liquid chromatography mass spectrometry in tandem mode (LC-MS/MS). For instance, enzymatically generated RvD_1_ was determined by LC-MS/MS analysis, and further evidence of its positive identification was obtained using HPLC-DAD to confirm the presence of a conjugated tetraene structure within RvD_1_ that is responsible for its characteristic triplet chromophore at a λ_max_ = 301 nm. A subsequent GC-MS analysis was performed after derivatizing RvD_1_ with diazomethane to its corresponding trimethylsilyl derivative [9,13]. In a similar way, actively phagocytosing polymorphonuclear neutrophils were converted to RvD_2_ and determined by LC-MS/MS analysis, followed by a subsequent GC-MS analysis of derivatized RvD_2_ to validate the LC-MS/MS determination [9]. The main drawback associated with HPLC-DAD is the potential coelution of isomeric resolvins with a similar spectrum, which may hinder their discrimination, while the most evident disadvantage of GC-MS is that is restricted to thermally stable volatile compounds, generally prepared by a time-consuming derivatization process [14,15,16]. 

Resolvins are commonly found in biological fluids and organs at extremely low concentrations, including peripheral blood, cerebral fluid, placenta, synovial fluids, urine, sputum, spleen, lymph nodes, cell cultures, and others [17]. As a result, successful extraction is essential to wash and clean up the sample, followed by drying up and reconstitution in a small volume to improve the concentration of the analyte. An overview of the literature indicated that solid phase extraction (SPE) is arguably the most popular extraction method for the analysis of resolvins in different kind of samples and LC-MS/MS the preferred quantitative technique for its outstanding sensitivity. A recent article has proposed a cumbersome methodology that combines liquid–liquid extraction (LLE) with chloroform and acetate-water buffered at pH 4 followed by µ-SPE with methanol-water buffered at pH 4 and a final LC-MS/MS analysis for the quantification of resolvins in human keratinocyte cell lysates to obtain recoveries at around 42 and 64% [18]. Unfortunately, SPE alone (not to mention when combined with LLE) is a time-consuming and complicated method that requires multiple steps and different solvents prior to LC-MS/MS analysis. Our current SPE protocol for the analysis of resolvins (RvD_1_, RvD_2_) in cell culture requires a total of seven different solvents (six solvents for SPE and one solvent for final reconstitution) [14]. Poor recovery and reproducibility, and insufficient cleaning of sample extracts are some of the drawbacks commonly associated with SPE [19]. Different SPE adsorbent materials and LLE have been compared to propose a suitable method for the extraction of RvD_1_ from human endothelial cells and further determination by LC-MS, and the best results were obtained using LLE with a solution of methanol containing the internal standard [20]. Although it seems a promising approach, this LLE protocol was validated incorrectly by using spiked plasma samples instead of human endothelial cells. Furthermore, methanol is generally used for protein removal, and therefore LLE with methanol for biological samples requires multiple and time-consuming centrifugation steps to ensure complete precipitation: for instance, centrifugation times of 45 min for metabolome [21] and 70 min for RvD_1_ analysis [20], followed by drying methanol with a stream of nitrogen, which is a lengthy operation prior to final reconstitution and LC-MS/MS analysis. 

Overall, sample preparation for the analysis of multiple compounds (e.g., resolvins) is the major bottleneck in analytical laboratories. The aim of this study is to propose a simple and rapid LLE procedure to quantify the temporal production of released resolvins (RvD_1_, RvD_2_, RvD_3_, RvD_4_, RvD_5_) into Leibovitz’s L-15 complete medium by head kidney cells from Atlantic salmon exposed to DHA and further liquid chromatography triple quadrupole mass spectrometry (LC-MS/MS). To our knowledge, this is the first validated LLE procedure for quantifying biosynthesized D-series resolvins using cell cultures. 

## 2. Results and Discussion

### 2.1. Optimal Concentration of Internal Standards

A 3^2^-factorial design, where the base 3 represents the number of concentration levels (low, medium, high) and the exponent 2 the number of factors (analyte and internal standard), was used to investigate whether the response factor (RF) remains constant at different internal standard (IS) concentrations over a fixed analytical range. The nine combinations of analytes and internal standards (3^2^) suggested by the factorial design (Table 1) were prepared by dissolving the analytical resolvins and internal standards in the L-15 medium. For instance, Experiment 2 represents an L-15 media solution containing all the analytes (RvD_1_, RvD_2_, RvD_3_, RvD_4_, RvD_5_) at a concentration level of 30 ng/mL and submitted to the extraction protocol by using a mixture of deuterated internal standards (RvD_1_-d_5_, RvD_2_-d_5_ and RvD_3_-d_5_) at 15 ng/mL each. Four blanks, consisting of L-15 medium containing 0, 15, 30 and 45 ng/mL of internal standards, were also prepared. The experiments in Table 1 were were prepared in triplicate and run in random order.

The RF for every resolvin at the different concentrations of IS were calculated at every experimental point in Table 1 by the expression RF *=* [IS]/[A] × (*y_A_*/*y_IS_*), where [A] and [IS] represent the analyte and IS concentrations and *y_A_* and *y_IS_* their corresponding signals, respectively. This RF approach has been published elsewhere in the selection of an optimal concentration of RvD_2_-d_5_ for extracting RvD_1_ and RvD_2_ from fish cells by SPE and further quantification using an LCMS ion-trap instrument [14]. The results are shown in Table 2.

A multiple range test, applied to determine significant differences (*p* < 0.05) between the calculated RF values, revealed that apart from RvD_5_, the RF remains constant for the concentrations 15 and 30 ng/mL of IS with coefficients of variations around 2%. Hence, 15 ng/mL of RvD_1_-d_5_ (for RvD_1_, RvD_4_ and RvD_5_), RvD_2_-d_5_ (for RvD_2_) and RvD_3_-d_5_ (for RvD_3_) that yields average RFs of 2.90 ± 0.05, 14.47 ± 0.33, 34.33 ± 0.61, 0.84 ± 0.01 and 1.01 ± 0.04 for RvD_1_, RvD_2_, RvD_3_, RvD_4_, and RvD_5_, respectively, was selected as the optimal concentration level to be used in connection with the proposed extraction protocol and for further quantitative analysis using LC-MS/MS. Although the RF values for RvD_5_ (1.10 ± 0.04, 1.01 ± 0.02, 1.19 ± 0.02 at 15, 30 and 45 ng/mL of IS, respectively), exhibited statistically significant differences, the RF for 15 ng/mL lay between 30 and 45 ng/mL, with a coefficient of variation (3.2%) similar to those reported elsewhere (4–13% at 1 ng/mL IS) for cell experiments using LCMS ^18^.

### 2.2. Analytical Validation

**Figure 1:**
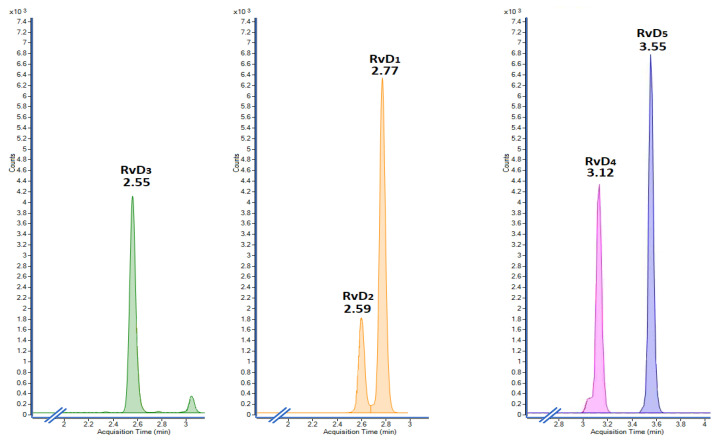
Extracted ion chromatograms, in increasing order of retention times, to indicate the selectivity of the analysis towards the five analyzed resolvins, after implementing the proposed LLE protocol.

The deuterated internal standards RvD_1_-d_5_, RvD_2_-d_5_ and RvD_3_-d_5_ were dissolved in acetonitrile (15 ng/mL each) and used to extract the resolvins from seven L-15 media preparations containing a mixture of five analytical resolvins (RvD_1_ to RvD_5_) at seven different concentrations (0, 1, 5, 15, 30, 45, and 50 ng/mL). The characteristic mass fragments of the five resolvins and three internal standards were extracted from the precursor ions in both unspiked (0 ng/mL) and spiked (1–50 ng/mL) L-15 media. The five resolvins were separated chromatographically, according to their retention times, including RvD_1_ and RvD_2_, which have the same precursor (*m*/*z* 375) and product (*m*/*z* 141) ions. The chromatographic elution order was RvD_3_ (2.55 min), RvD_2_ (2.59 min) RvD_1_ (2.77 min), RvD_4_ (3.12 min), and RvD_5_ (3.55 min). The extracted ion chromatograms (EIC) revealed that the proposed extraction protocol allows for detecting unequivocally the resolvins with negligible background interferences (Figure 1), and therefore the analyses were regarded as highly selective towards the five resolvins.

The chromatographic peak area ratios RvD_1_/RvD_1_-d_5_, RvD_2_/RvD_2_-d_5_, RvD_3_/RvD_3_-d_5_, RvD_4_/RvD_1_-d_5_ and RvD_5_/RvD_1_-d_5_ were calculated and plotted against the analytical concentrations to compute the function $y_{A}/y_{IS}=\varphi [A]+\beta$ and obtain the various calibration parameters displayed in Table 3. 

The degree of linearity of the calibrations was provided by both the regression coefficients (*R*^2^) and the Fisher test, defined as the quotient between the lack-of-fit and the pure error variances (*F_exp_* in Table 3). In general, the five resolvins were linear over the studied range of concentrations, as reflected in Table 3, where the *R*^2^ values (between 0.994 and 0.999) indicate that a high proportion of the variance of the calculated *y_A_*/*y_IS_* signals are explained by the analytical concentrations [A] in the proposed regression models. This conclusion is also supported by the *F_experimental_* values for the five calibration models that were lower than the critical value of 2.958 for 5 and 14 degrees of freedom at the 95% confidence level (Table 3). The LOD (0.028–0.059 ng/mL) and LOQ (0.074–0.180 ng/mL) were within the range of previously reported values for cell cultures [22], and were considered appropriate for all resolvin species. In the present work, the LOQ for resolvins in L-15 complete media by using an Agilent 6495 triple quadrupole are similar to (and in some cases better than) those reported in pure standards by using Sciex QTRAP 6500 [23,24]. For instance, the referred Quadrupole/QTRAP (present/[24]) values for RvD_1_, RvD_2_, RvD_3_, RvD_4_ and RvD_5_ are 0.127/0.05, 0.5/0.086, 0.097/0.05, 0.074/0.1 and 0.180/0.1, respectively. Based on the matrix complexity, namely the present L-15 medium versus the pure standard [24], the LOQ values of the present research can be regarded as remarkable. In addition, the proposed extraction protocol in conjunction with the triple quadrupole spectrometer is an outstanding strategy, considering the widespread consensus that QTRAP delivers better data than quadrupole systems [25]. The recovery of the method, expressed as the ratio between found and nominal concentrations (100 × [A]_found_/[A]_nominal_) was higher than 95% in all cases, as described in Table 3.

### 2.3. Analysis of Released Resolvins in L-15 Media by Head Kidney Cells 

The proposed LLE protocol and further LC-MS/MS quantification was implemented to study the induced production of resolvins by salmon head kidney cells with and without exposure to exogenous DHA. The production of resolvins was expressed in ng/mL and measured at 6, 12 and 24 h (Table 4). The levels of resolvins in decreasing order of concentration were RvD_4_ > RvD_2_ > RvD_3_ > RvD_1_ > RvD_5_ and RvD_4_ > RvD_3_ > RvD_2_ > RvD_1_ > RvD_5_ in control and DHA, respectively. These levels agree with previously reported results that were obtained by using SPE and LC-MS/MS to estimate the production of RvD_1_ and RvD_2_ by salmon liver cells, and where it was suggested that the production of RvD_4_ was preferred over RvD_1_ and RvD_2_ after exposing the cells to different polyunsaturated fatty acids, including DHA [14].

**Figure 2:**
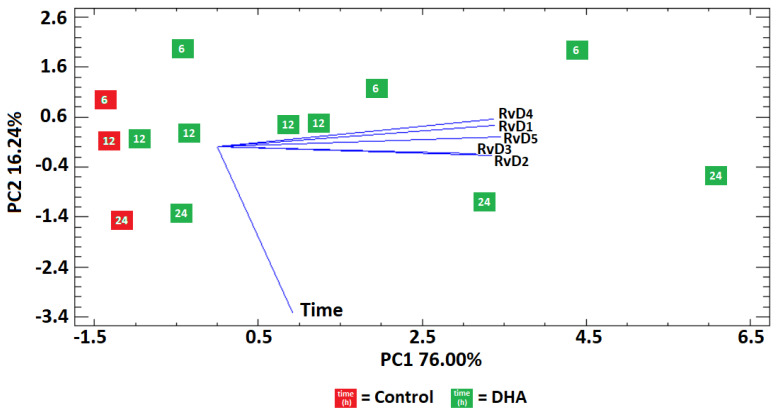
Principal component analysis of the released resolvins in L-15 media by head kidney cells with and without exposure to DHA, after implementing the proposed LLE protocol.

A principal component analysis (PCA) indicated that 76.00% of the total data variability was explained by the concentrations of resolvins in control and DHA groups, and 16.24% was explained by the different times (Figure 2). 

The control and DHA groups were clearly discriminated along the PC1 axis and characterized by negative and positive scores along this axis, respectively. The degree of overlapping within the control and within the DHA group was higher in the former than in the latter group. For instance, the scores for the control at 6 or 12 or 24 h appeared as three separated clusters along PC2 (red squares), while for the DHA the time clusters were separated along PC2 (green squares), but they were widely spread along PC1, indicating a higher dispersion of the DHA data. The PCA also showed that the highest concentrations of resolvins were associated with the DHA group, suggesting that exogenous DHA promoted the production of resolvins. The vectors, time and concentrations of resolvins, were orthogonal; therefore, the changes in concentrations of RvD_1_, RvD_2_, RvD_3_, RvD_4_ and RvD_5_ were independent of the time. This lack of correlation between time and concentration was confirmed by studying the within and between variances at the three selected times for every resolvin. The estimated *p*-values were not significant (*p* > 0.05) for any type of resolving, either in the control or in the DHA group.

**Figure 3:**
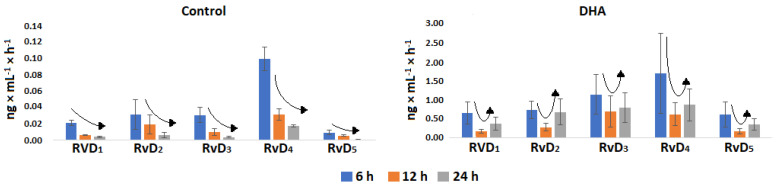
Concentration/time ratio for the different resolvins in the control and DHA group. The former group shows a continuous decrease in production and the latter a plausible regulated production by a circadian clock.

The concentration/time relationship was computed from Table 4, and the results revealed a continuous decrease in production over time for the five analyzed resolvins in the control group (Figure 3). In contrast, the DHA group showed higher production at subjective dawn (6 h) and subjective midnight (24 h) than subjective midday (12 h) for all resolvins (Figure 2), suggesting the presence of a circadian clock that may impose a 24 h rhythmicity on the head kidney cells to process the production of resolvins from the added 50 μM of DHA. 

The direct influence of circadian rhythms on resolvin production remains unexplored. However, considering the present findings, it is plausible that there exists an underlying resolvin/15-LOX immune regulating clock that controls the production of resolvins from endogenous DHA. This observation is supported by the results from different studies that have observed higher levels of COX-1 and inflammatory prostaglandins (PGE_2_ and PGF_2α_) at midnight than at midday, and concluded that some immunological functions are controlled by circadian responses from a prostaglandin/COX-1 system [26]. Similarly, anti-inflammatory prostaglandin 15d-PGJ_2_ has been identified as an entrainment factor aligned with circadian oscillations [27].

## 3. Materials and Methods

### 3.1. Reagents

Resolvin D1 (RvD_1_, 95%), resolvin D2 (RvD_2_, 95%), resolvin D3 (RvD_3_, 95%), resolvin D4 (RvD_4_, 95%), resolvin D5 (RvD_5_, 95%), deuterated resolvin D1 (RvD_1_-d_5_, 95%), deuterated resolvin D2 (RvD_2_-d_5_, 95%) and deuterated resolvin D3 (RvD_3_-d_5_, 95%). Acetonitrile (99.8%) and formic acid (98%) were purchased from Sigma-Aldrich (St. Louis, MO, USA). 2-propanol (HPLC grade, 99.9%) from Merck (Darmstadt, Germany). Chloroform (HPLC grade, 99.8%) was obtained from Merck (Darmstadt, Germany). A Millipore Milli-Q system was used to produce ultra-pure water 18 MΩ (Millipore, Milford, CT, USA). Cis-4,7,10,13,16,19-docosahexaenoic acid (DHA, ≥98%) were purchased from Sigma-Aldrich (Oslo, Norway). Leibovitz’s L-15 medium was from Sigma-Aldrich (St. Louis, MO, USA). Fetal bovine serum (FBS, cat# 14-801F) was from BioWhittaker (Petit Rechain, Belgium). The glutaMaxTM 100× (Gibco-BRL, cat# 35056) was from Gibco-BRL (Cergy-Pontoise, France).

### 3.2. Head Kidney Cells

For each fish, the head kidneys were directly sampled and added to PBS at 5 °C and then cut with scissors and squeezed through a 40 µM Falcon cell strainer. The cells were transferred to tubes and centrifuged in a Hettich Zentrifugen, 320 R, at 400× *g* for 5 min at 4 °C. The cell pellets were resuspended in PBS and layered carefully on top of equal amounts of diluted Percoll in a density of 1.08 g/mL. The tubes were centrifuged at 800× *g* for 30 min at 4 °C. The cell layer in the interface containing the head kidney leukocytes was collected and the cells were pelleted by centrifugation, 400× *g* for 5 min at 4 °C. An additional washing step in PBS was performed. The cells were counted using a Bürker chamber and 0.4% trypan blue solution, and the viability was above 85%.

### 3.3. Cell Cultures

A L-15 complete (cL-15) medium was supplemented with 10% foetal bovine serum (FBS), 2% pen/strep and 2% glutamax^TM^100×, and used to prepare cL-15 solutions containing DHA that was diluted to a concentration of 50 μM and a control solution containing ethanol (the solvent used to dissolve the DHA). Approximately 1 × 10^7^ salmon head kidney cells were cultured into each well (control and DHA). The cell culture plates were incubated in a normal atmosphere incubator (Sanyo Electric Company Ltd. Osaka, Japan) at 9 °C for 6, 12, and 24 h, under dark conditions. The two suspensions of cells (control and DHA) were prepared in pentaplicate. The head kidney cells were centrifuged at 50× *g* for 5 min at 4 °C, and the medium collected and stored at −80 °C until extraction, followed by the LC-MS/MS analysis.

### 3.4. Optimal Concentrations of the Internal Standards 

A 3^k^ factorial design, where 3 represents the number of concentration levels (low, medium, high) and k the number of factors (analyte and internal standard), was used to study variations in the response factor (RF) when the concentrations of both the internal standards (IS) and the analytical resolvins varied between 15 and 45 ng/mL. The optimal IS concentrations should yield a stable RF over the explored analytical range. 

### 3.5. Extraction Protocol

The extraction protocol has been described elsewhere for the determination of arachidonic and eicosapentaenoic acid metabolites from the LOX pathway [28], with some minor modifications. Briefly, two successive aliquots of acetonitrile (500 μL) containing the mixture of internal standards at the concentration levels indicated in Table 1 and chloroform (500 μL) were added successively into an Eppendorf tube containing 200 μL of the mixture of resolvins, at the concentration levels of every experiment in Table 1. The Eppendorf tube was vortex-mixed for 30 s (Bandelin RK 100 ultra mixer, Berlin, Germany) and centrifuged at 1620× *g* for 3 min (Eppendorf AG centrifuge, Hamburg, Germany), the top phase was removed, and the extraction procedure repeated in the remaining phase using acetonitrile without internal standards and chloroform. After removing the chloroform phase, the remaining solution was vacuum-dried at room temperature (Labconco vacuum drier system, Kansas, MO, USA), diluted to 50 μL with methanol, transferred to an autosampler vial, and submitted to LC-MS/MS analysis. 

### 3.6. Analytical Performance

The parameters used to assess the analytical performance of the extraction method in conjunction with LC-MS/MS were selectivity, limit of detection (LOD), limit of quantification (LOQ), calibration range and recovery. The selectivity of the method was evaluated by comparing the chromatograms obtained after injection of L-15 medium samples with and without the analytes. The calibration curves for the resolvins in the L-15 medium were prepared between 0 and 50 ng/mL and extracted as described above, by using the optimal concentration of internal standards suggested by the 3^k^ factorial design. The linearity was judged by computing both the variance ratio of the lack-of-fit to pure error and the coefficient of regression, as suggested by the Analytical Method Committee [29] and the International Council for Harmonisation guidance for the validation of analytical procedures [30]. The ratio of the standard deviation (σ) to the slope (φ) of the regression curves for every resolvin was used to determine the LOD (3.3 × σ/φ) and LOQ (10 × σ/φ), as described elsewhere [31]. The percentage of recovery was assessed by comparing the degree of agreement between the experimental and nominal concentrations, as acknowledged by the ICH [30,31]. 

### 3.7. Liquid Chromatography Mass Spectrometry 

An Agilent ultra-high performance liquid chromatography (UHPLC), coupled to a 6495 QQQ triple quadrupole (Agilent Technologies, Waldbronn, Germany) with an electrospray ionization (ESI) interface and iFunnel ionization, was used to quantify the eicosanoids. The UHPLC system was equipped with a Zorbax RRHD Eclipse Plus C18, 95Å, 2.1 × 50 mm, 1.8 µm chromatographic column. The mobile phase delivered at 0.4 mL/min in gradient mode consisted of ultra-pure water with 0.1% formic acid (solution A) and an equal-volume mixture of acetonitrile and methanol with 0.1% formic acid (solution B). The solvent gradient was as follows: solution A was reduced from 60 to 5% from 0.00 to 4.00 min, kept at 5% between 4.00 and 5.50 min, increased to 60% between 5.50 and 5.51 min and kept at 60% between 5.51 and 10.00 min. Mass spectrometric detection was performed by multiple reactions monitoring (MRM) in negative mode. The monitored transitions in percentage of ion counts (%) were: *m*/*z* 375 → 141 for RvD_1_ and RvD_2_; *m*/*z* 375 → 147 for RvD_3_; *m*/*z* 375 → 101 for RvD_4_; *m*/*z* 359 → 199 for RvD_5_; *m*/*z* 380 → 141 for RvD_1_-d_5_; *m*/*z* 380 → 141 for RvD_2_-d_5_; and *m*/*z* 380→147 for RvD_3_-d_5_. The ESI parameters were gas temperature (120 °C), gas flow rate (19 L/min), nebulizer pressure (20 psi), sheath gas temperature (300 °C), sheath gas flow (10 L/min), capillary voltage (3500 V) and nozzle voltage (2000 V). The integration of the chromatograms was performed using the MassHunter Qualitative Navigator software (version 10.0). The levels of resolvins were estimated by means of the internal standards, and expressed in ng/mL units.

### 3.8. Statistics

Statgraphics Centurion XV Version 15.2.11 (StatPoint Technologies, Inc., Warrenton, VA, USA) was used for the statistical analyses. 

## 4. Conclusions

This is the first validated liquid–liquid extraction method for resolvins released by head kidney cells from Atlantic salmon in cL-15 media with further quantification by LC-MS/MS using the internal standard calibration method. The small amount of sample (200 μL), the low solvent consumption, the fast extraction times, the high sample throughput (40–50 samples/day) and LOD and LOQ similar to those reported by using SPE and pure standards are important features that make the present approach highly attractive for evaluating the production of resolvins by cell cultures challenged by polyunsaturated fatty acids, such as DHA. 

In light of the present findings, it is plausible that production of resolvins by head kidney cells from endogenous DHA is controlled in part by circadian responses.

## Figures and Tables

**Table 1:** Proposed 3^2^-factorial design to select the optimal concentrations of internal standards.

Experiment	Resolvin [A] (ng/mL)	Internal Standard [IS] (ng/mL)
1	15	15
2	30	15
3	45	15
4	15	30
5	30	30
6	45	30
7	15	45
8	30	45
9	45	45

**Table 2:** Calculated response factors (RF) after implementing the 3^2^-factorial design to select the optimal amount of deuterated internal standard for the liquid–liquid extraction of resolvins from L-15 cell culture media. The RF values are expressed as average ± standard error.

[IS]	RvD_1_-d_5_	RvD_2_-d_5_	RvD_3_-d_5_	RvD_1_-d_5_	RvD_1_-d_5_	RvD_1_-d_5_	RvD_2_-d_5_	RvD_3_-d_5_	RvD_1_-d_5_	RvD_1_-d_5_	RvD_1_-d_5_	RvD_2_-d_5_	RvD_3_-d_5_	RvD_1_-d_5_	RvD_1_-d_5_
	15 ng/mL					30 ng/mL					45/ng/mL				
[A] (ng/mL)	RvD_1_	RvD_2_	RvD_3_	RvD_4_	RvD_5_	RvD_1_	RvD_2_	RvD_3_	RvD_4_	RvD_5_	RvD_1_	RvD_2_	RvD_3_	RvD_4_	RvD_5_
15	2.70	12.41	33.74	0.79	1.00	2.85	12.81	34.63	0.83	0.95	3.32	16.68	39.22	1.00	1.16
15	2.57	13.78	31.24	0.79	0.92	2.77	14.39	32.76	0.87	0.93	3.35	16.73	38.37	0.99	1.15
15	3.04	15.80	36.96	0.93	1.10	2.85	14.05	34.03	0.87	0.96	3.27	16.00	39.58	1.01	1.22
30	3.06	15.07	36.22	0.85	1.07	3.03	14.97	32.95	0.89	1.03	3.12	15.41	35.83	0.95	1.25
30	2.94	14.01	32.97	0.80	1.08	3.24	16.35	37.58	0.98	1.14	3.23	15.43	35.06	0.91	1.26
30	2.98	15.17	33.63	0.86	1.26	3.09	15.24	32.57	0.95	1.00	3.18	16.22	38.42	0.94	1.32
45	2.92	14.63	34.31	0.84	1.11	2.82	14.53	32.44	0.82	1.01	2.92	14.15	34.87	0.83	1.16
45	2.97	14.58	33.71	0.84	1.11	2.87	14.33	34.57	0.87	0.98	2.92	14.86	32.89	0.90	1.09
45	2.96	14.80	36.16	0.82	1.24	2.60	13.35	31.01	0.80	1.05	2.97	14.57	32.29	0.82	1.14
AVG	2.90 ± 0.05	14.43 ± 0.33	34.10 ± 0.61	0.84 ± 0.01	1.08 ± 0.04	2.94 ± 0.06	14.58 ± 0.35	33.94 ± 0.63	0.89 ± 0.02	1.00 ± 0.02	3.17 ± 0.06	15.69 ± 0.31	36.78 ± 0.91	0.94 ± 0.02	1.20 ± 0.02
%CV	1.86	2.27	1.78	1.75	3.25	2.14	2.37	1.85	2.21	2.14	1.79	1.95	2.47	2.48	2.01

%CV = coefficient of variation.

**Table 3:** Analytical performance parameters. The linearity is judged by considering simultaneously the closeness of *R*^2^ to the unity and the comparison of *F_experimental_* against the tabulated *F_critical_* = 2.958 for 5 and 14 degrees of freedom at the 95% confidence level.

Resolvin (0–50 ng/mL)	Slope (φ)	Intercept (*β*)	*R* ^2^	*F_experimental_*	LOD	LOQ	Recovery (%)
RvD_1_	0.197	−0.063	0.997	0.526	0.042	0.127	98.2 ± 1.3
RvD_2_	0.982	−0.235	0.999	0.342	0.028	0.086	99.5 ± 1.1
RvD_3_	2.304	−0.318	0.998	0.142	0.032	0.097	99.5 ± 0.9
RvD_4_	0.055	0.002	0.999	0.123	0.024	0.074	99.8 ± 1.2
RvD_5_	0.077	−0.048	0.994	0.624	0.059	0.180	96.9 ± 2.3

**Table 4:** Temporal production of resolvins by head kidney cells from Atlantic salmon (*Salmon salar*) exposed to docosahexaenoic acid.

	Time	RvD_1_	RvD_2_	RvD_3_	RvD_4_	RvD_5_
Group	(hours)	Concentrations (ng/mL)				
Control	6	0.127 ± 0.022	0.190 ± 0.109	0.186 ± 0.055	0.601 ± 0.090	0.055 ± 0.018
	12	0.074 ± 0.009	0.238 ± 0.141	0.119 ± 0.049	0.382 ± 0.082	0.069 ± 0.009
	24	0.094 ± 0.014	0.154 ± 0.079	0.092 ± 0.018	0.419 ± 0.031	0.017 ± 0.003
DHA	6	3.918 ± 1.798	4.412 ± 1.386	6.849 ± 3.156	10.231 ± 6.342	3.696 ± 2.022
	12	1.388 ± 0.404	2.574 ± 1.143	5.212 ± 2.328	4.737 ± 1.710	1.395 ± 0.361
	24	4.391 ± 2.103	8.095 ± 4.129	9.583 ± 4.682	10.439 ± 5.121	4.256 ± 1.815

## Data Availability

All the used data have been provided in the text.
